# Engineering functionalized multi-phased silicon/silicon oxide nano-biomaterials to passivate the aggressive proliferation of cancer

**DOI:** 10.1038/srep12141

**Published:** 2015-07-20

**Authors:** P. Premnath, B. Tan, K. Venkatakrishnan

**Affiliations:** 1 Department of Mechanical and Industrial Engineering, Ryerson University, Toronto, Canada, M5B 2K3; 2 Department of Aerospace Engineering, Ryerson University, Toronto, Canada, M5B 2K3

## Abstract

Currently, the use of nano silicon in cancer therapy is limited as drug delivery vehicles and markers in imaging, not as manipulative/controlling agents. This is due to limited properties that native states of nano silicon and silicon oxides offers. We introduce nano-functionalized multi-phased silicon/silicon oxide biomaterials synthesized via ultrashort pulsed laser synthesis, with tunable properties that possess inherent cancer controlling properties that can passivate the progression of cancer. This nanostructured biomaterial is composed of individual functionalized nanoparticles made of a homogenous hybrid of multiple phases of silicon and silicon oxide in increasing concentration outwards from the core. The chemical properties of the proposed nanostructure such as number of phases, composition of phases and crystal orientation of each functionalized nanoparticle in the three dimensional nanostructure is defined based on precisely tuned ultrashort pulsed laser-material interaction mechanisms. The amorphous rich phased biomaterial shows a 30 fold (95%) reduction in number of cancer cells compared to bulk silicon in 48 hours. Further, the size of the cancer cells reduces by 76% from 24 to 48 hours. This method exposes untapped properties of combination of multiple phases of silicon oxides and its applications in cancer therapy.

The advent of nanotechnology has introduced nanoparticles and other nanostructures to the forefront of cancer therapy. However, the use of nanoparticles is limited as drug delivery carriers and markers in targeted therapy^1^,^2^; and nanostructures are exclusively researched as biosensors for cancer detection^3^,^4^. Further, biocompatible elements like nano silicon have only been researched as drug delivery agents, potential markers for imaging or diagnostic chips^5^,^6^,^7^. Therefore, it is observed that the applications for silicon nanoparticles are limited due to usage in its pure form and absence of modulated phase and composition of phases of pure silicon and silicon oxides.

Extensive studies revealed the limited scope of silicon and silicon oxides in cancer therapies. Silicon based nanoparticles have been applied to the field of imaging. Erogogbo *et al.* generated biocompatible luminescent silicon quantum dots by laser pyrolysis followed by etching until a required size is obtained^6^. These quantum dots were used as labels for pancreatic cancer cells. In addition to imaging, detection is another area of cancer therapy where silicon nanoparticles have been employed. For example, silicon nanowires owing to its electrical and biocompatible properties were used for biomarker detection^8^. Silicon nanoparticles are also employed as photosensitizers against cancer cells via photodynamic therapy. For instance, Xiao *et al.* formulated silicon nanoparticles via electrochemical etching from a single silicon crystal^9^.They were able to show 45% cancer cell death compared to 25% cell death where no nanoparticles were employed. It is observed that in all the studies, silicon has only been employed as a nanoparticle in its pure form. Further, the use of an external agent such as radiation is necessary.

Research in the area of drug delivery has also employed silicon/silicon oxide nanoparticles, focusing on porous silica nanoparticles as potential drug carriers. This is owing to several advantages that mesoporous silica offers like high stability, being chemically versatile and biocompatibility^7^. Further, it is a good specimen for controlled release of drugs^10^. For instance Meng *et al.* developed mesoporous silica nanoparticles via sol-gel process that can be loaded with Doxorubicin and P-Glycoprotein siRNA that would aid in cancer cell killing^11^. These nanoparticles have dual characteristics of eluting chemotherapeutic drugs as well as siRNA capable of reducing drug resistance. Similarly, Rosenholm *et al.* synthesized a hybrid mesoporous silica nanoparticle with poly(ethylene imine) and different target moieties^12^. Sol-gel technique was employed in their study in addition to surface polymerization to synthesize these nanoparticles for targeted therapy. Zhang *et al.* synthesized mesoporous silica nanoparticles that target cancer cells selectively due to the addition of folate targeting agents^13^. Therefore, similar to the use of radition in concurrence with silicon nanoparticles in imaging, additional drugs like doxorubicin are required for use with silicon nanoparticles in therapy. Another limiting factor has been the pure state of silicon nanoparticles. To the best of our knowledge, there have been no studies that explore the combination of a homogenous phase of silicon and silicon oxides as potential cancer controlling agents in a three dimensional nanostructure. The generation of these phases deactivates the inherent proliferative nature of silicon.

We report the generation of multi-phased silicon/silicon oxide nano biomaterials that are a fibrous aggregation of functionalized nanoparticles via ultrashort pulsed laser synthesis. The functionalized nanoparticles form a core-shell like structure with a combination of homogenous phases of silicon and silicon oxides. The unique aspect of ultrashort pulsed laser synthesis method lies in the fact that each individual functionalized nanoparticle can be modified in its chemical and physical form. When the ultrashort pulsed laser, with pulse to pulse separation time between micro and nanoseconds, interacts with the surface of silicon, it vaporizes silicon. Subsequently, these species nucleate, coalesce and aggregate to form three dimensional nanoparticle aggregates. Each of these functionalized nanoparticles in the three dimensional nano biomaterial are made up of a crystalline silicon core, and is enveloped by an increasing concentration of silicon oxide. The phase as well as size of the functionalized nanoparticle is controlled by altering the plume dynamics, controlling the surface and plume temperature during ultrashort pulsed laser-material interaction. It is observed that the multi-phased nano biomaterial deactivates the proliferative nature of silicon and reduces cancer cell numbers compared to bulk silicon. The growth of cervical cancer cells on this functionalized multi-phased nano biomaterial exhibit remarkable properties of cell proliferation control. A 95% (30 fold) decrease in cancer cells is observed on amorphous rich nanofibers produced at the shortest pulse duration. The health of the cell is also quantified by assessing cell size that showed drastic reduction in cancer cell size at the end of 48 hours on nanostructured silicon. We attribute this reduction to the potential internalization of nanoparticles and/or encapsulation of cells by nanofibers. Previous studies of similar nano materials has exclusively characterized its proliferative properties^14^,^15^,^16^.On the contrary, this study presents selective cell death based on the homogenous fusion of multiple phases of silicon and silicon oxide.

## Materials and Methods

### Generation of nano-functionalized multi-phased nano biomaterial

Silicon wafers are modified in a single step using ultrashort pulses with pulse separation time between micro and nanoseconds. Undoped silicon wafers <111> with a thickness of 500 μm (University Wafers, USA) are cut into 2 × 2 cm samples. Samples are washed with de-ionized (DI) water and ethanol and rinsed in DI water again. After air drying, the samples are irradiated using a diode pumped, Yb-doped femtosecond laser system (Clark-MXR Inc. IM-PULSE Series Ultrashort Pulse laser) at 4,8 and 26 MHz. The laser irradiates the samples in an array of lines at distances between 100 μm to 2 mm. The speed at which the laser irradiates and the power of the laser is maintained at 10 mm/s and 15 W respectively. The laser pulse width or peak power plays an important role and is varied between 214fs (short pulse), 714fs (medium pulse) and 1428fs (long pulse) corresponding to 8.76MW, 2MW and 1.31MW peak power respectively. All the parameters are controlled by a computer to facilitate precision and accuracy.

### Characterization of functionalized multi-phased nano biomaterial

Scanning electron microscopy (SEM) (Hitachi S 5200) is performed to characterize the morphology of the multi-functionalized nanostructures. The samples are loaded onto aluminum stands and gold coated for SEM. Energy-dispersive X-ray spectroscopy (EDX) is carried out to determine the elemental composition of irradiated silicon. Micro-raman spectroscopy as well as X-Ray photon spectroscopy (Thermo scientific K-alpha) is used further to study surface composition quantitatively and qualitatively. X-ray diffraction(Siemens D5000 conventional theta/2theta diffractometer) is used as a secondary tool to confirm elemental composition.

### Cancer cell- nano biomaterial interaction

Cervical cancer cells (HeLa, ATCC, USA) are employed to qualitatively and quantitatively study cancer controllability. The cells are grown in DMEM/F12 medium supplemented with 10%fetal bovine serum and 1% Pen-strep. The nanostructured silicon is sterilized under UV light for 20 minutes. Subsequently, the samples are placed in petri dishes and HeLa cells at a density of 10^5^ cells/ml, totaling 3 ml per dish are seeded onto the nanostructured substrate. The petri dishes are placed in an incubator for 24 and 48 hours. Each 2 × 2 cm substrate consists of 0.5 × 0.5 cm^2^ of nano-biomaterial.

For SEM imaging, after the incubation period the samples are fixed in 2% glutaraldehyde in 0.1 M sodium cacodylate buffer pH 7.3 for an hour. Next, the samples are immersed in 0.1 M sodium cacodylate buffer with 0.2 M sucrose pH 7.3 for 20 minutes. This is followed by dehydration in increasing concentrations of alcohol for 20 minutes each is followed. The samples are then critical point dried. SEM imaging is performed at 5 kV acceleration voltage and magnification between 100X and 10,000X.

For fluorescence microscopy the samples are first fixed in methanol free paraformaldehyde followed by incubation with milk to prevent non-specific binding. To stain the actin and cytoskeleton, the samples are incubated with Alexa fluor 488(Life Technologies) followed by DAPI (Life Technologies) to stain the nucleus. The samples are studied using a fluorescence microscope (Nikon, Canada).

### Statistics

All experiments are carried out in triplicates and the data points are averages unless otherwise mentioned. The error bars indicate standard deviations.

## Results and Discussion

### Synthesis of nano-functionalized multi-phased biomaterial

**Figure 1:**
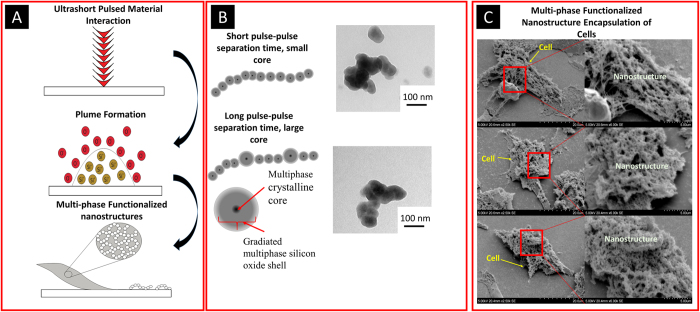
(**A**) Illustration of ultrashort pulse laser-material interaction (**B**) Illustration of mechanism of formation of functionalized multi-phased silicon/silicon oxide nanostructures (**C**) Mode of cell encapsulation by fibers.

The interaction of ultrashort laser pulses results in the formation of multi-phased nano biomaterial that is an aggregation of functionalized nanoparticles forming nanofibers, comprising of silicon as well as silicon oxides, (Fig. 1) with a crystalline core enveloped by increasing concentrations of silicon oxides. The formation and composition of these functionalized multi-phased nano biomaterials is determined by varying plume dynamics. When a single laser pulse strikes the surface of silicon, the region is heated followed by heat dissipation through the lattice. The number of atoms that evaporate from the surface after single pulse interaction is low^17^. However, in this research study, similar to current industrial trends, multiple pulses are employed. The frequency of the multiple pulses is precisely controlled between 4 and 26 MHz. This frequency determines the separation time between the pulses which varies from micro to nanoseconds. When the pulse to pulse separation is reduced, there is not enough time for the heat to dissipate and is concentrated at the zone of laser-material interaction. Another parameter to consider with respect to heat dissipation, is the speed of the laser which in this case is maintained at 10 mm/s. Therefore, a single spot receives multiple pulses for 1 ms. Tavangar *et al.* showed that the density of ablated material is dependent on the pulse to pulse separation time (frequency of pulses) as well as the laser-material interaction time using the following equation^18^


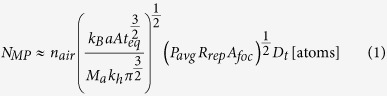


Where *R*_*rep*_ is the frequency of pulses and *D*_*t*_ is the laser material interaction time. It is evident from the above equation that the pulse to pulse separation time plays a critical role in the density of atoms that is ablated from the surface. The ablated density is directly proportional to the density of functionalized multi-phased nano biomaterials that are synthesized on the surface. In this study, it is evident that the density of functionalized multi-phased nano biomaterials plays an important role in controlling the growth of cells, which will be elaborated in the following sections.

**Figure 2:**
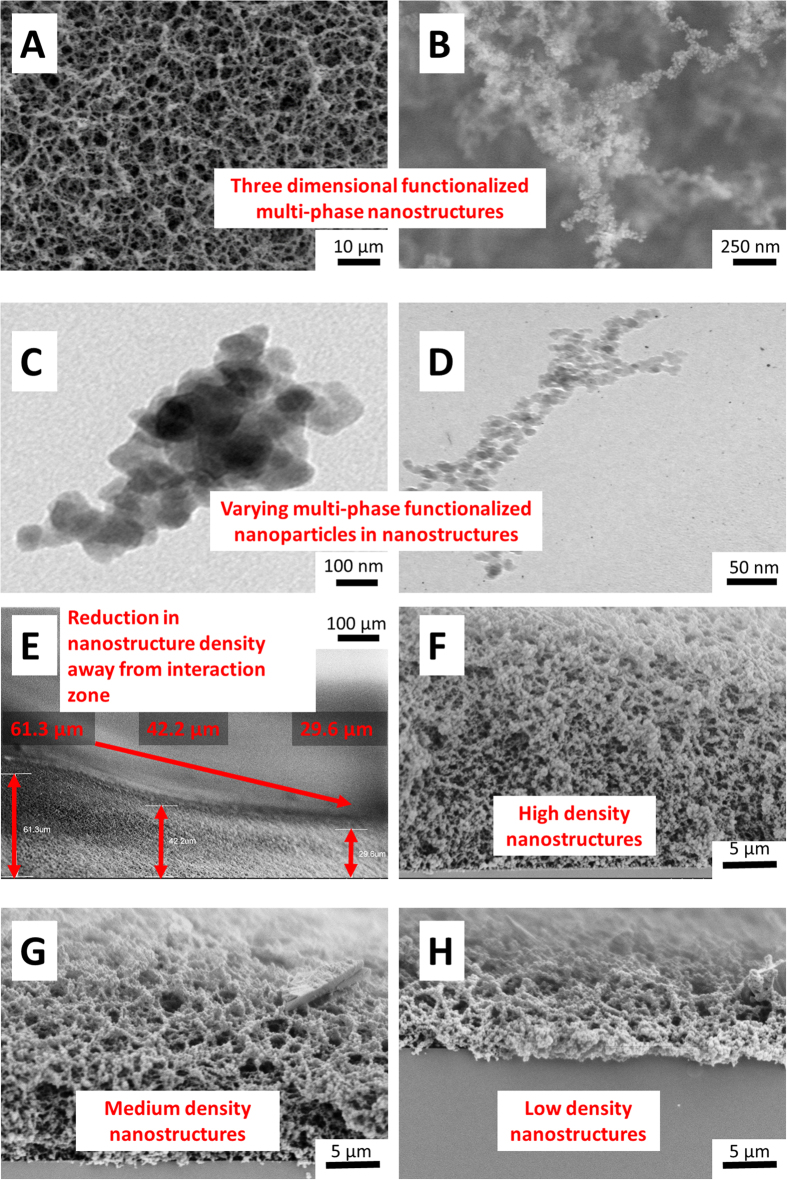
(**A**,**B**) SEM images of functionalized multi-phased nanostructured biomaterial(**C**,**D**) TEM images showing various functionalized nanoparticle sizes (**E**–**H**) SEM micrographs of varying nanostructure density of functionalized multi-phased biomaterial from area of laser-material interaction and away.

The synthesis of nano-functionalized multi-phased biomaterials can be explained by mechanisms in the plume. Specifically, based on the size of functionalized nanoparticle it is apparent that vapor condensation mechanism is responsible for formation. The cooling of the plume is initiated by diffusion in ambient air. The ablated matter is considered to be in a supercritical fluid state owing to above threshold fluence of the laser^19^. At the end of a laser pulse, ions interact to form molecules which collide due to thermal motion. When the temperature falls, the formation of functionalized nanoparticles begins^20^. The formation of functionalized nanoparticles is followed by coalescence and coagulation. And finally aggregation of coagulates takes place. This aggregation may exist between previously aggregated species or an aggregated species and atoms. While the process of formation of functionalized nanoparticles in the multi-phased nano biomaterial remains the same, the size of the functionalized nanoparticles varies according to frequency of incoming pulses (Fig. 2(A–D)). It is evident, that the functionalized nanoparticle size on average decreased with an increase in number of pulses. Sivayoganathan *et al.* showed a unique bimodal distribution of silicon nanoparticles at lower frequencies^21^. Even though the average size of the nanoparticles stayed constant, lower frequencies had a small fraction of nanoparticles that were larger. This could be attributed to the presence of neutral species in addition to ionized species in the plume.

The SEM images also provide an insight into the density of the functionalized multi-phased nano biomaterial (Fig. 2(E–H). It is observed that zones away from the area of laser material interaction have proportionally lower density of functionalized multi-phased nano biomaterial. In addition to the density, the morphology is also significantly different as we move further away, the multi-phased nano biomaterial changes to fragments of agglomerated functionalized nanoparticles.

### Composition of nano-functionalized multi-phased nano biomaterial

**Figure 3:**
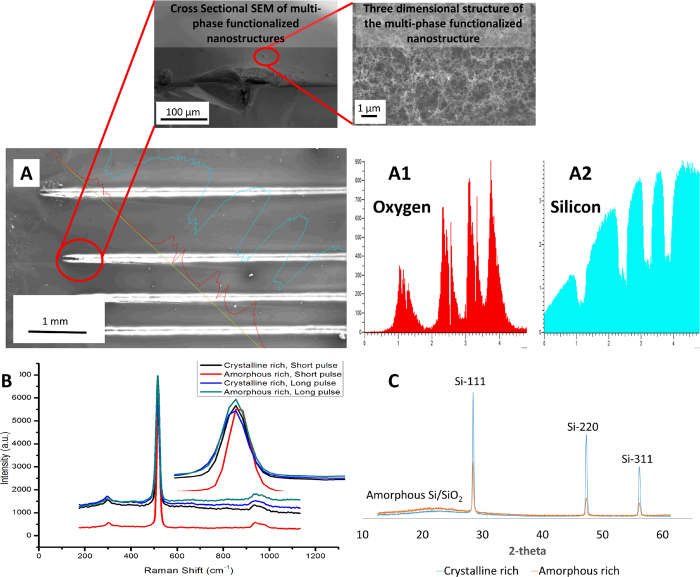
(**A**) SEM micrograph depicting ultrashort pulsed laser-material interaction zones. The magnified images show the functionalized multi-phased nanostructured biomaterial density at point of interaction and away (A1) Oxygen content (A2) Silicon content (**B**) Raman spectroscopy graphs at varying pulse durations and different phase concentrations of the multi-phased nanostructured biomaterial (**C**) XRD graphs at different phase concentrations multi-phased nanostructured biomaterial.

Subsequent to the study of morphology of the functionalized nanoparticles in the multi-phased nano biomaterial, the material composition is analyzed. The study is conducted based on EDX, XRD and Raman analysis. EDX shows the presence of oxygen in areas deposited with functionalized multi-phased nano biomaterial (Fig. 3(A,A1. EDX is used only as a preliminary qualitative tool of analysis due to sharp peaks of substrate silicon distorting data. Raman analysis was performed to qualitatively assess the different phases present (Fig. 3(B)). The image shows the presence of various phases of silicon in the functionalized multi-phased nano biomaterial. The Raman micrograph reveals the broadening of peaks at 26 MHz which may is potentially due to the introduction of amorphous silicon. Further, the peak signifying silicon has been shifted from 520 cm^−1^on bulk to 516^−1^ cm at all parameters. This is an indication of change in particle sizes which can be calculated by^22^


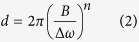


Where d is the crystal diameter and is calculated to be approximately 4.43 nm when  = 2 cm^−1^nm^2^and Δω is the shift in peak. Further analyses on the peaks reveal irregularities in the width of the peaks. This is attributed to defects in grains being produced in the nanoparticles. This is also a potential indication on polycrystalline silicon. Another interesting fact is also observed in the Raman micrographs. The peaks are broader towards higher Raman shifts for short pulse and broader towards lower Raman shifts for long pulse. This disparity is due to the difference in defects being introduced at different pulse durations.

To further study the composition of this functionalized multi-phased nano biomaterial, XRD is employed (Fig. 3(C)). XRD reveals the presence of three distinct crystal structures of silicon, Si (111), (220) and (311). These crystal structures are observed at all parameters. However, the presence of amorphous oxides depends on the frequency of pulses. It is seen that as the pulse to pulse separation time is reduced or the frequency of pulses is increased there is a corresponding increase in amorphous oxides. It is seen that the functionalized multi-phased nano biomaterial contain randomly oriented micro crystallites of silicon. This nature of randomly oriented micro crystalline structures could be attributed to varying plume mechanisms. The collisions of species with different crystal orientations may result in such attributes^19^.

The mode of formation of the functionalized multi-phased nano biomaterial is described based on different plume mechanisms. At long pulse to pulse separations, there is high crystal concentration compared to amorphous oxides. This is due to slow cooling of liquid crystal droplets. At shorter pulse to pulse separation times, there is not enough time for the species to cool and hence the introduction of amorphous oxides takes place. We hypothesize that the amorphous oxides are formed as an external ‘shell’ surrounding the crystalline silicon. At shorter pulse to pulse separation times, more species are ejected into the plume. Further, ionization of gases around the plume also takes place. Therefore, there is a higher chance of silicon and oxygen collisions further promoting the formation of amorphous silicon oxide shells^23^. The size of the nanoparticles also depends upon similar tendencies of the nucleation of species. Higher density promotes more nucleation sites encouraging smaller nanoparticles. A lower density of species causes fewer nucleation sites with bigger nanoparticles.

### Cancer cell manipulation

#### Adherent nano-functionalized multi-phased nano biomaterial

**Figure 4:**
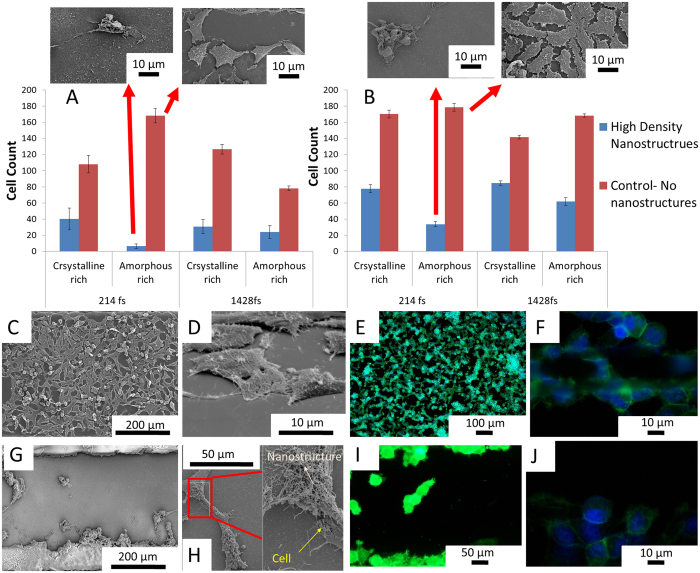
Number of cells on different phases of functionalized multi-phased nano biomaterial as well as different pulse generated nanostructures. Comparison is made between cells on high density functionalized multi-phased nano biomaterial and control or virgin silicon at (**A**) 24 hours and (**B**) 48 hours. (**C**,**D**) SEM images of HeLa cells on control or virgin silicon (**E**,**F**) Fluorescence microscopy images of HeLa cells on control or virgin silicon (**G**,**H**) SEM images of HeLa cells on high density functionalized multi-phased nano biomaterial (**I**,**J**) Fluorescence microscopy images of HeLa cells on high density functionalized multi-phased nano biomaterial. All images are of cell growth at 48 hours.

Surface-bound functionalized multi-phased nano biomaterial adhere strongly to the surface of silicon owing to high temperature formation in the plume. These functionalized multi-phased nano biomaterial form a three dimensional matrix which is interlinked and randomly oriented. In contradiction to other research studies that present cell proliferation on nanofibrous structures, this study shows reduction in the rate of cell proliferation^24^. Silicon substrates with their functionalized multi-phased nano biomaterial ultrasonically removed are considered as control samples. As evident in Fig. 4, there is approximately a 95% reduction in the growth of cells on amorphous rich functionalized multi-phased nano biomaterial fabricated at short pulse. Moreover, there is a reduction in the number of cells on any surface that has surface-bound functionalized multi-phased nano biomaterial. The disparity in reduction of cells could be attributed to encapsulation of cells in the amorphous rich functionalized multi-phased nano biomaterial fabricated at short pulse. The cell proliferation and health is further analyzed based on the density and phase of fibers.

#### Cell proliferation: Role of density and phase of functionalized multi-phased nano biomaterial

The synthesis of functionalized multi-phased nano biomaterial causes differences in its phases and density. As mentioned previously, the density of functionalized multi-phased nano biomaterial closest to the zone of laser material interaction is significantly higher and will consist of high temperature and high pressure phases. To analyze the effects of density on the growth characteristics of HeLa cells, cell count as well as cell health are assessed at both 24 and 48 hours. It is observed that at 24 hours, more number of cells have attached to the high density fibers compared to low density fibers. There is no clear indication of differences in cell attachment at different pulse durations. However, while comparing cell attachment at 24 hours, it is evident that the number of cells that attached at 4 MHz (Crystalline rich) are significantly higher than those that attached at 26 MHz (Amorphous rich)(Fig. 4(A,B). We attribute this unique tendency of cell growth to the morphology and composition of the functionalized multi-phased nano biomaterial. After 48 hours of cell growth, there is evidence of change in cell attachment characteristics. There is a complete reversal of cell attachment with respect to density. There are significant increases in cell growth on low density fibers compared to high density fibers. This is comparable at all parameters. However, it is still apparent that amorphous rich phases presents lower cell attachment compared to crystalline rich phases. An interesting phenomenon occurs here where percentage cell proliferation is completely reversed between amorphous rich and crystalline rich phases. At 48 hours, it is clear that the rate of proliferation is consistently higher for amorphous rich phases compared to crystalline rich phases.

Therefore, it is seen that cells are affected by multiple parameters that are each controllable by varying laser parameters. An increase in density of nano-functionalized multi-phase biomaterial (high density nanostructures) reflects the two aspects, an increase in nanostructures itself as well as an increase in high temperature phases. Nisbet *et al.* reviewed the response of neutrites on different densities of electrospun fibers^25^. They found a correlation between the distances of fibers at various densities dictated cell growth. Since our functionalized multi-phased nano biomaterial are in fact composed of individual nanoparticles, we postulate that we increase the surface area for protein adsorption and initial cell attachment. At 24 hours, cells preferentially attach to areas of higher protein adsorption, which in this case is the high density of nanostructures^26^. After initial attachment, it is hypothesized that the cells are trapped or encapsulated in the fibers and hence their growth potential is severely hampered. At the same time, the cells that are growing on low density of nanofibers are not encapsulated by fibers but rather the fibers forms points of adhesion. Therefore, there is a marked increase in cell proliferation in low density of fibers over a period of 48 hours. It is been found that cancer cells are inherently predisposed to migrating through fibers to move to other areas and metastasize^27^. Due to this intrinsic feature, they are more likely to be encapsulated by high density functionalized multi-phased nano biomaterial.

The phase of the functionalized multi-phased nano biomaterial itself has a significant effect on the attachment characteristics of cells. From the XRD, it is clear that frequency of pulses or pulse to pulse separation time has a major influence on the crystalline vs. amorphous nature of functionalized multi-phased nano biomaterial. Further, at 26 MHz, due to less pulse to pulse separation time more silicon species are formed in the plume. Consequently, we see an increase in the density of the functionalized multi-phased nano biomaterial. However, the functionalized multi-phased nano biomaterial that are synthesized at 26 MHz are not strongly bonded and due to this reason encapsulation of cells occurs. There have been previous studies that show that cells prefer a crystalline surface to attach on to compared to an amorphous surface^28^.

**Figure 5:**
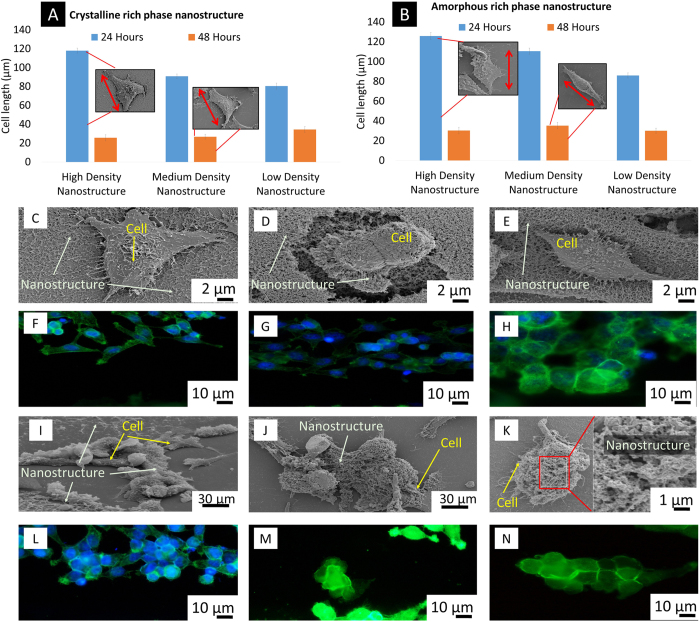
(**A**) The length along the major axis of HeLa at 24 and 48 hours on crystalline rich phases on varying densities of functionalized multi-phased nano biomaterial(**B**) The length along the major axis of HeLa at 24 and 48 hours on amorphous rich phases on varying densities of functionalized multi-phased nano biomaterial (**C**–**E**) SEM images of cell growth at 48 hours on crystalline rich phases of functionalized multi-phased nano biomaterial (**F**–**H**) Fluorescence microscopy images of cell growth at 48 hours on crystalline rich phases of functionalized multi-phased nano biomaterial(**I**-**K**) SEM images of cell growth at 48 hours on amorphous rich phases of functionalized multi-phased nano biomaterial(**L**-**N**) Fluorescence microscopy images of cell growth at 48 hours on amorphous rich phases of functionalized multi-phased nano biomaterial.

In addition to studying the number of cells present, the health of the cells of the cells is also considered (Fig. 5). To quantify the health of the cells, the length along their major axis is calculated^29^. It is that evident that at 24 hours, irrespective of the density, the cells were longer on nano-functionalized multi-phased biomaterials that were amorphous rich. Cells also preferred high density compared to low density functionalized multi-phased nano biomaterial. A remarkable phenomena occurs after 48 hours. The length of the cells is sharply reduced to lesser than 50% of its original length for both amorphous and crystalline rich phases.

**Figure 6:**
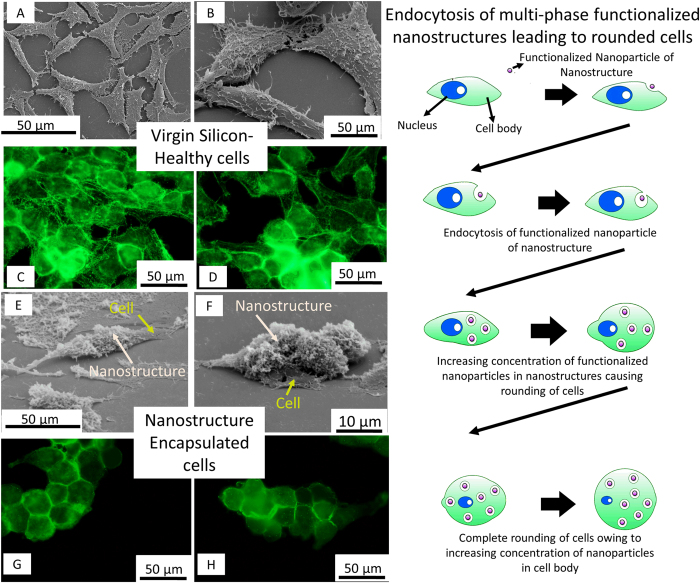
Mechanism of cell death. (**A**,**B**) SEM images of HeLa on control virgin silicon after 48 hours (**C**,**D**) Fluorescence microscopy images of HeLa on control virgin silicon after 48 hours (**E**,**F**) SEM images of HeLa on amorphous rich functionalized multi-phased nano biomaterial after 48 hours (**G**,**H**) Fluorescence microscopy images of HeLa on amorphous rich functionalized multi-phased nano biomaterial after 48 hours. The illustration on the right elucidates a potential cell death mechanism.

A difference in density of the functionalized multi-phased nano biomaterial causes a trend in the way the cells attach to them. An increase in density reflects larger surface area for the cells to attach to^30^. Further, Zhao *et al.* proved that nanostructures induce higher amounts of collagen secretion as well as protein adsorption^31^. Therefore, at 24 hours cells are flatter on denser functionalized multi-phased nano biomaterial. Amorphous rich phases showed better cell adhesion owing to two factors- particle size as well as higher concentration of functionalized nanoparticles. The drastic reduction in size that is observed at 48 hours maybe explained by passive ingestion of functionalized nanoparticles in the 48 hour period (Fig. 6). Similar results were seen in a study conducted by Jin *et al.* where the fibroblasts showed a reduction in size when the concentration on nano titanium was increased in the solution^32^. The process of internalization is described based on lysosome activity in the cells.

To study the cellular mechanism and adhesion, fluorescence microscopy is performed. Figure 5(F–H,L–N) present virgin silicon and functionalized multi-phased nano biomaterial and their interaction with HeLa. On control virgin silicon, cells grow randomly with well defined, flattened cytoskeleton. Focal adhesion points are observed at random points in the cell. These focal adhesion points are the interaction points of cells and the substrate. Stress fibers are cues for the cells based on mechanical signals from the substrate^33^. The density and organization of the stress fibers are an indication of the stress being transmitted by the substrate. The stress fibers on control silicon are randomly arranged. Interaction of HeLa cells with functionalized multi-phased nano biomaterial, both adherent and deposited presents cells that are rounded and drastically reduced in size. Stress fibers are completely absent in cells growing on functionalized multi-phased nano biomaterial silicon. Since stress fibers are an integral part of cell adhesion, it can be concluded that cells are apoptotic. The edges of the cells appear brighter signifying adhesion points.

#### Cell Health: Exploring the nucleus

**Figure 7:**
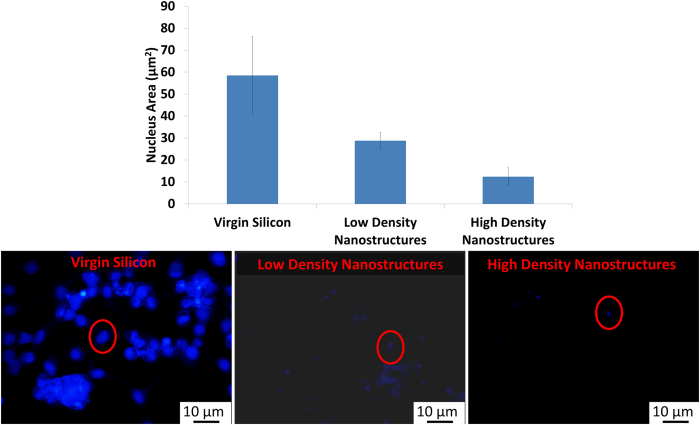
Measuring nucleus over low and high density nano-functionalized multi-phased biomaterial. The graph shows reduced nucleus size at high density. The circled areas point to a representative nucleus.

Figure 7 is clearly indicative of differences in nuclei, it is evident that the nucleus is well rounded on cells growing on control virgin silicon as opposed to the nucleus of cells grown on functionalized multi-phased silicon. The elliptical or polarized shape of the nucleus is owing to actin cytoskeletal forces^34^. In addition, the size of the nucleus also significantly reduces with increasing functionalized multi-phased nano biomaterial density. It is hypothesized that a reduction in nucleus size may induce signaling that lead to apoptosis. Further, altered nucleus size may indicate changes in chromatin organization and gene expression^35^.

## Conclusion

This manuscript reports functionalized silicon nano biomaterials that are a homogenous combination of multiple phases of silicon and silicon oxides. Due to its limited scope, the use of pure silicon/silicon oxide based nano biomaterials in cancer therapy has been limited to drug delivery carriers, cancer marker detectors and as photosensitizers in photodynamic therapy. Through functionalized silicon/silicon oxide based nano biomaterials we present novel drug-like properties that are capable of controlling the growth of cancer. Ultrashort pulsed laser synthesis is employed to fabricate nano-functionalized multi-phased nano biomaterial directly on the surface of silicon. The three dimensional nano biomaterial is composed of individual functionalized nanoparticles with a crystalline silicon core enveloped by increased concentration of silicon/silicon oxide shell. The unique aspect of this mode of synthesis is precise controllability of the number of phases, concentration of phases and size of the crystalline core enveloped amorphous surrounding. This is owing to precise control over the surface temperature and plume temperature that alters the ultrashort pulsed laser-material interaction mechanism. This functionalized multi-phased nano biomaterial deactivates the proliferative nature of silicon and passivates cancer cell growth owing to the unique blend of multiple phases of silicon and silicon oxide. The culture of HeLa cells on the functionalized multi-phased nano biomaterial shows a critical dependence of cancer cell proliferation on topography as well as material chemistry of functionalized multi-phased nano biomaterial. It is observed that there is a 30 fold (95%) reduction in the number of HeLa cells after 48 hours on amorphous rich phases of multi-phased nanostructures. The nucleus reduced in size significantly on high density of functionalized multi-phased nano biomaterial. Two hypothesis for reduction in number of cells is put forth- endocytosis of functionalized multi-phased nano biomaterial and encapsulation of cells by functionalized multi-phased nano biomaterial. Therefore, this nano-functionalized multi-phased nano biomaterial diminishes cell proliferation unlike other research studies on pure silicon nanostructures that show the cell proliferation. The novelty of this study lies in the method of synthesis as well as engineering nanostructured biomaterials. To the best of our knowledge, this is the first study to show generation of multi-phased nanostructured biomaterials with cancer controlling properties synthesized via ultrashort pulsed laser interaction. Ultrashort pulsed laser synthesis of functionalized multi-phased nano biomaterial holds immense potential in a myriad of cancer therapy applications such as in implants, diagnostic chips and markers in targeted therapy and opens avenues for the application of various nano forms of silicon in addition to precisely varying number of and composition of phases of both silicon and silicon oxides.

## Additional Information

**How to cite this article**: Premnath, P. *et al.* Engineering functionalized multi-phased silicon/silicon oxide nano-biomaterials to passivate the aggressive proliferation of cancer. *Sci. Rep.* **5**, 12141; doi: 10.1038/srep12141 (2015).
